# Temporal Trends in Cardiovascular Hospital Discharges Following a Mass Chlorine Exposure Event in Graniteville, South Carolina

**DOI:** 10.3389/fpubh.2019.00112

**Published:** 2019-05-08

**Authors:** Ashley V. Howell, John E. Vena, Bo Cai, Daniel T. Lackland, Lucy A. Ingram, Andrew B. Lawson, Erik R. Svendsen

**Affiliations:** ^1^Department of Public Health Sciences, Medical University of South Carolina, Charleston, SC, United States; ^2^Arnold School of Public Health, University of South Carolina, Columbia, SC, United States

**Keywords:** chlorine poisoning, chemical hazards release, disasters, hypertension cardiovascular disease, toxic gas exposure, disaster epidemiology

## Abstract

**Background:** On January 6, 2005, a train derailed in Graniteville, South Carolina, releasing nearly 60,000 kg of toxic chlorine gas. The disaster left nine people dead and was responsible for hundreds of hospitalizations and outpatient visits in the subsequent weeks. While chlorine gas primarily affects the respiratory tract, a growing body of evidence suggests that acute exposure may also cause vascular injury and cardiac toxicity. Here, we describe the incidence of cardiovascular hospitalizations among residents of the zip codes most affected by the chlorine gas plume, and compare the incidence of cardiovascular discharges in the years leading up to the event (2000–2004) to the incidence in the years following the event (2005–2014).

**Methods:** De-identified hospital discharge information was collected from the South Carolina Revenue and Fiscal Affairs Office for individuals residing in the selected zip codes for the years 2000 to 2014. A quasi-experimental study design was utilized with a population-level interrupted time series model to examine hospital discharge rates for Graniteville-area residents for three cardiovascular diagnoses: hypertension (HTN), acute myocardial infarction (AMI), and coronary heart disease (CHD). We used linear regression with autoregressive error correction to compare slopes for pre- and post-spill time periods. Data from the 2000 and 2010 censuses were used to calculate rates and to provide information on potential demographic shifts over the course of the study.

**Results:** A significant increase in hypertension-related hospital discharge rates was observed for the years following the Graniteville chlorine spill (slope 8.2, *p* < 0.001). Concurrent changes to CHD and AMI hospital discharge rates were in the opposite direction (slopes −3.2 and −0.3, *p* < 0.01 and 0.14, respectively). Importantly, the observed trend cannot be attributed to an aging population.

**Conclusions:** An unusual increase in hypertension-related hospital discharge rates in the area affected by the Graniteville chlorine spill contrasts with national and state-level trends. A number of factors related to the spill may be contributing the observation: disaster-induced hypertension, healthcare services access issues, and, possibly, chlorine-induced susceptibility to vascular pathologies. Due to the limitations of our data, we cannot determine whether the individuals who visited the hospital were the ones exposed to chlorine gas, however, the finding warrants additional research. Future studies are needed to determine the etiology of the increase and whether individuals exposed to chlorine are at a heightened risk for hypertensive heart disease.

## Introduction

In the early morning hours of January 6, 2005, a freight train carrying three tank cars full of liquid chlorine derailed near the center of Graniteville, South Carolina, USA. One of the chlorine tanks ruptured in the accident, releasing nearly 60,000 kg of chlorine gas (1–3), which quickly overwhelmed workers on the graveyard shift at the nearby Avondale Mills textile plant. As the dense gas plume spread and settled in the valley, thousands of residents were exposed to the poisonous gas. A mass evacuation of the surrounding communities lasted for several days while emergency crews decontaminated the area, and photographs of the aftermath captured bleached and dead foliage and wildlife (4, 5). Ultimately, the disaster left nine people dead and was responsible for 72 hospitalizations and more than 840 emergency room and outpatient visits in the subsequent days (1, 2, 6, 7). Beyond the initial acute effects of chlorine gas, the results of public health-related screenings of exposed Graniteville residents demonstrated that the harmful pulmonary effects of exposure continued for months after the event (7, 8).

A substantial body of research has characterized the immediate effects of chlorine in animal models and humans (9–11). Following acute chlorine exposure, individuals are at risk for pulmonary edema, respiratory failure, and death by suffocation (9, 12). Consistent with the literature, studies of patient hospitalizations following the Graniteville train derailment confirmed the occurrence of frequent respiratory symptoms, and autopsies of the nine dead listed the primary cause of death as asphyxia or acute respiratory failure (1, 2). While the primary effects of acute exposure occur in the respiratory tract, a growing body of evidence suggests chlorine can also cause vascular injury and cardiac toxicity resulting in increased long-term cardiovascular risks (5, 13–15). In Graniteville, autopsies found eight of the nine dead suffered from cardiomegaly(7), and a study of lung function in the Avondale mill workers conducted 7 to 9 years after the event noted that 12% of subjects were unable to complete spirometry tests due to uncontrolled elevated blood pressure.

In addition to the health risks posed by chlorine gas exposure, a second threat to the cardiovascular health of the Graniteville community is “disaster-induced hypertension” (16). A number of studies conducted in the aftermath of natural and anthropogenic disasters have documented an increase in cardiovascular-related problems following the events. For example, following the 2011 earthquake and tsunami in Japan, Aoki et al. identified an increase in all types of cardiovascular disease events that lasted between 2 and 4 weeks and occurred in regions where radiation contamination was low (17). Similarly, researchers found a rise in cerebrovascular and heart disease hospitalizations and deaths after the World Trade Center Disaster in 2001 (18), and a 3-fold increase in acute myocardial infarctions in the 6 years following Hurricane Katrina (19). Activation of the sympathetic nervous system is postulated to be the primary driver of the phenomenon, likely due to disrupted circadian rhythms and psychological stress (20). Such activation of the sympathetic nervous system may, in turn, increases heart rate, blood pressure variability, and platelet activation (20).

Despite the documented risks to heart health, no studies to-date have investigated cardiovascular disease incidence in populations exposed to chlorine gas. Moreover, little is known about the long-term health effects of acute chlorine exposure, particularly cardiovascular disease. This study had two aims: (1) to describe the incidence of cardiovascular hospitalizations in the years surrounding the Graniteville chlorine spill, and (2) to determine whether there were significant changes in cardiovascular hospitalizations observed (at the population level) following the event.

## Methods

### Exposure Assessment

In a 2015 article, Jani et al. modeled the dispersion of the Graniteville chlorine plume using the Hazard Prediction and Assessment Capability (HPAC) modeling system, which incorporates topographical and meteorological data to optimize accuracy (3). This model was later validated with exposure indicator data (21). Using a geographical overlay of the published plume model, we selected seven zip codes that were most affected by the chlorine plume for our study area: 29801, 29816, 29828, 29829, 29834, 29850, and 29851. The plume area lies within Aiken County, SC and includes about 45,000 residents.

### Outcome Data

De-identified hospital discharge information was collected from the South Carolina Revenue and Fiscal Affairs Office (SC RFA) for individuals residing in the selected zip codes. Data were available for the years 2000 through 2014 and captured discharges of the Graniteville residents from hospitals located in other states in addition to discharges from South Carolina hospitals. To protect patient confidentiality visit dates were limited to the year of hospital discharge, and patient age was provided in 5-year age categories. Records associated with the following ICD-9 codes were included in our analysis: acute myocardial infarction (AMI), ICD-9 code 410; hypertension, ICD-9 codes 401-404; and coronary heart disease (CHD) excluding AMI, ICD-9 codes 411-414, and 429.2. Selected records included both primary and secondary cardiovascular diagnoses, and individual patients were included more than once if s/he was discharged from the hospital multiple times with cardiovascular diagnoses. We were unable to assess repeated hospitalizations with this de-identified design. Admission records with multiple types of cardiovascular-related diagnoses were included in the statistics for each type of cardiovascular-related complaint (AMI, hypertension, or CHD).

### Demographic Data

Population demographic information was collected for each zip code from the 2000 and 2010 U.S. Censuses (22). To assess whether shifting demographic characteristics played a role in the rate of cardiovascular hospital discharges, the population composition for 2000 and 2010 were compared. We were specifically interested in age, sex, and race variables, as cardiovascular disease is influenced by these factors.

### Statistical Analysis

We used Microsoft Access (Redmond, WA) to store and query study data, and SAS 9.2 software (SAS Institute; Cary, NC) to calculate descriptive statistics and perform regression modeling. Summary statistics were calculated for each cardiovascular diagnosis by sex, payer type (Medicare/Medicaid, private insurance, or self-pay), visit type, race, and age categories. Mean annual rates of total and diagnosis-specific hospital discharges were calculated and stratified by sex and by age group. As dates of hospital visits were limited to the year of discharge, the rates calculated for 2005 included 5 days of “unexposed” time due to the chlorine disaster occurring on January 6, 2005.

We used a population-level interrupted time series design to compare trends in hospital discharge rates over time for the pre- and post-chlorine spill periods. To model trends in annual hospital discharge rates, linear regression with autoregressive error correction was used to adjust for serially correlated residuals (PROC AUTOREG in SAS) (23, 24). For each specified diagnosis, slopes and intercepts of the regression models were estimated for the time period leading up to the chlorine spill (2000–2004) and for the years following the event (2005–2014). In time series modeling, temporally variant covariates are most relevant (25). Therefore, population composition from the 2000 and 2010 censuses were compared to assess significant demographic shifts over the course of the study period. Any remaining unmeasured invariant covariates cannot confound these models because their effects would not vary between the pre- and post- exposure periods.

### Ethical Considerations

Before receipt, all data were de-identified and aggregated to the yearly average by the SC RFA. This study was reviewed and approved by the Medical University of South Carolina's Institutional Review Board and the SC RFA data oversight committee. To further protect the privacy of area residents, the data presented are aggregated by zip codes or aggregated over the entire study area.

## Results

We identified 13,275 hospital discharge records associated with one or more of the specified cardiovascular diagnostic codes between 2000 and 2014 in the study zip codes. Of all cardiovascular-related discharges, 5,669 (42.7%) included a primary or secondary diagnosis of CHD, 10,698 (80.6%) referenced hypertension diagnoses, and 1,046 (7.9%) cited an AMI diagnosis (Table 1). Men comprised the minority of hypertension-related discharges (42.2%), but represented the majority of visits for coronary heart disease (56.6%) and acute myocardial infarction (54.4%). Most of the patients seen were between the ages 65 and 74 (21.7%), followed closely by those aged 55 to 64 (21.3%) and 45 to 54 (19.6%). Only 1.1% of the hospital discharge records for our selected outcomes were for patients under the age of 25. Of those, most were related hypertension.

**Table 1:** Characteristics of cardiovascular hospital discharges by diagnosis (primary or secondary) among Graniteville-area residents for the years 2000–2014.

		**HTN**		**CHD**		**AMI**		**All**	
		**N**	**%**	**N**	**%**	**N**	**%**	**N**	**%**
Sex	Male	4,556	42.2	3,266	56.6	570	54.4	6,170	45.9
	Female	6,234	57.8	2,505	43.4	478	45.6	7,278	54.1
Visit type	Inpatient	5,314	49.3	3,461	60.0	1,015	96.9	6,771	50.4
	ED visits	4,488	41.6	822	14.2	29	2.8	4,834	36.0
	Outpatient services	988	9.2	1,488	25.8	4	0.4	1,843	13.7
Payer type	Medicare	5,876	54.5	3,679	63.8	632	60.3	7,553	56.2
	Medicaid	1,184	11.0	373	6.5	54	5.2	1,330	9.9
	Self-Pay/Indigent Care	1,434	13.3	328	5.7	111	10.6	1,584	11.8
	Insurance	2,296	21.3	1,391	24.1	251	24.0	2,981	22.2
Race^*^	White	5,669	52.6	4,441	77.1	793	75.8	7,910	58.9
	Black	4,976	46.2	1,235	21.4	237	22.7	5,354	39.9
	Other	134	1.2	87	1.5	16	1.5	170	1.3
Age	Under 25	143	1.3	3	0.1	0	0.0	144	1.1
	25–34	486	4.5	44	0.8	6	0.6	507	3.8
	35–44	1,093	10.1	256	4.4	72	6.9	1,220	9.1
	45–54	2,197	20.4	908	15.7	190	18.1	2,641	19.6
	55–64	2,299	21.3	1,339	23.2	203	19.4	2,868	21.3
	65–74	2,164	20.1	1,636	28.4	229	21.9	2,911	21.7
	75–84	1,668	15.5	1,216	21.1	216	20.6	2,237	16.6
	85 and older	740	6.9	369	6.4	132	12.6	920	6.8

HTN, hypertension; CHD, coronary heart disease excluding AMI; AMI, acute myocardial infarction; ED, emergency department.

* Missing race information (n = 14).

About half of the hypertension and CHD hospital discharge records were for inpatient visits (49.3% and 60.0%, respectively), while the almost all of the AMI-related discharges were for inpatient visits (96.9%). Emergency Department visits comprised 14.2% of the CHD visits 41.6% of hypertension visits, and 2.8% of AMI visits. The remaining records were for outpatient services, which included imaging, observation, and outpatient surgical procedures (9.2, 25.8, and 0.4% for hypertension, CHD, and AMI diagnoses, respectively).

**Figure 1:**
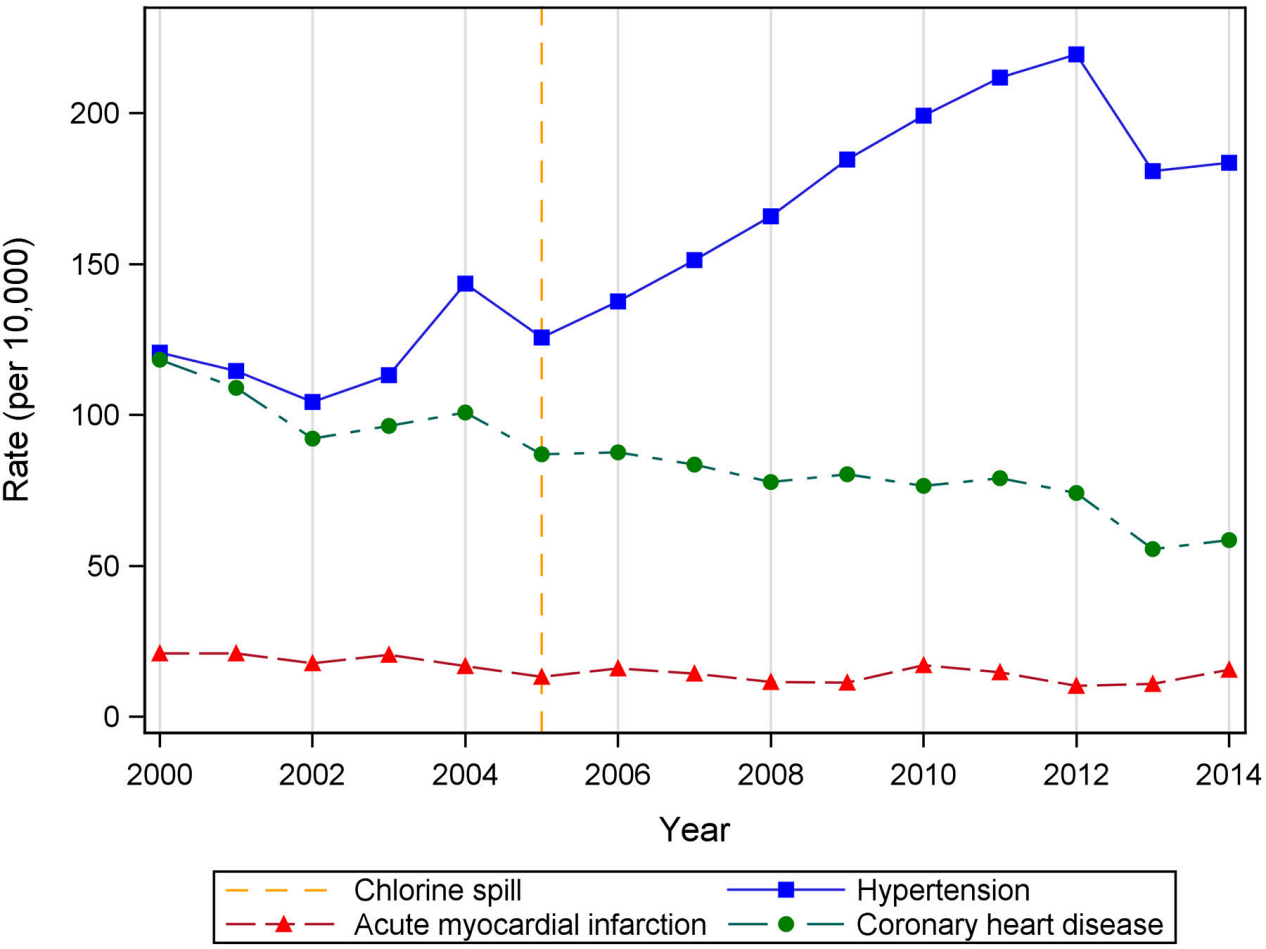
Annual rates of cardiovascular hospital discharges by diagnosis (primary or secondary) among Graniteville-area residents, 2000–2014.

Figure 1 illustrates the annual admission rates for our diagnoses of interest with the year of the chlorine accident noted with a dashed line to facilitate easy comparison of the pre/post disaster time periods. Upon examination of the annual trends in hospital discharge rates (Figure 1), we saw a pronounced increase in hypertension hospital discharges over time in contrast to small changes in CHD and AMI discharges. Rates of hypertension discharges rose from 120.7 per 10,000 in 2000 to 183.6 in 2014, an increase of 52%. At its peak in 2012, the rate was 219.5 per 10,000 which represents almost a 2-fold increase over the 2000 rate. A minor decrease in AMI rates was present over the study period: from 21.0 discharges per 10,000 persons to 15.6 per 10,000. This represents a 28% decrease. Rates of CHD-related hospital discharges experienced a marked declined over the study period, dropping from 118.3 per 10,000 persons in 2000 to 58.6 per 10,000 persons in 2014.

**Figure 2:**
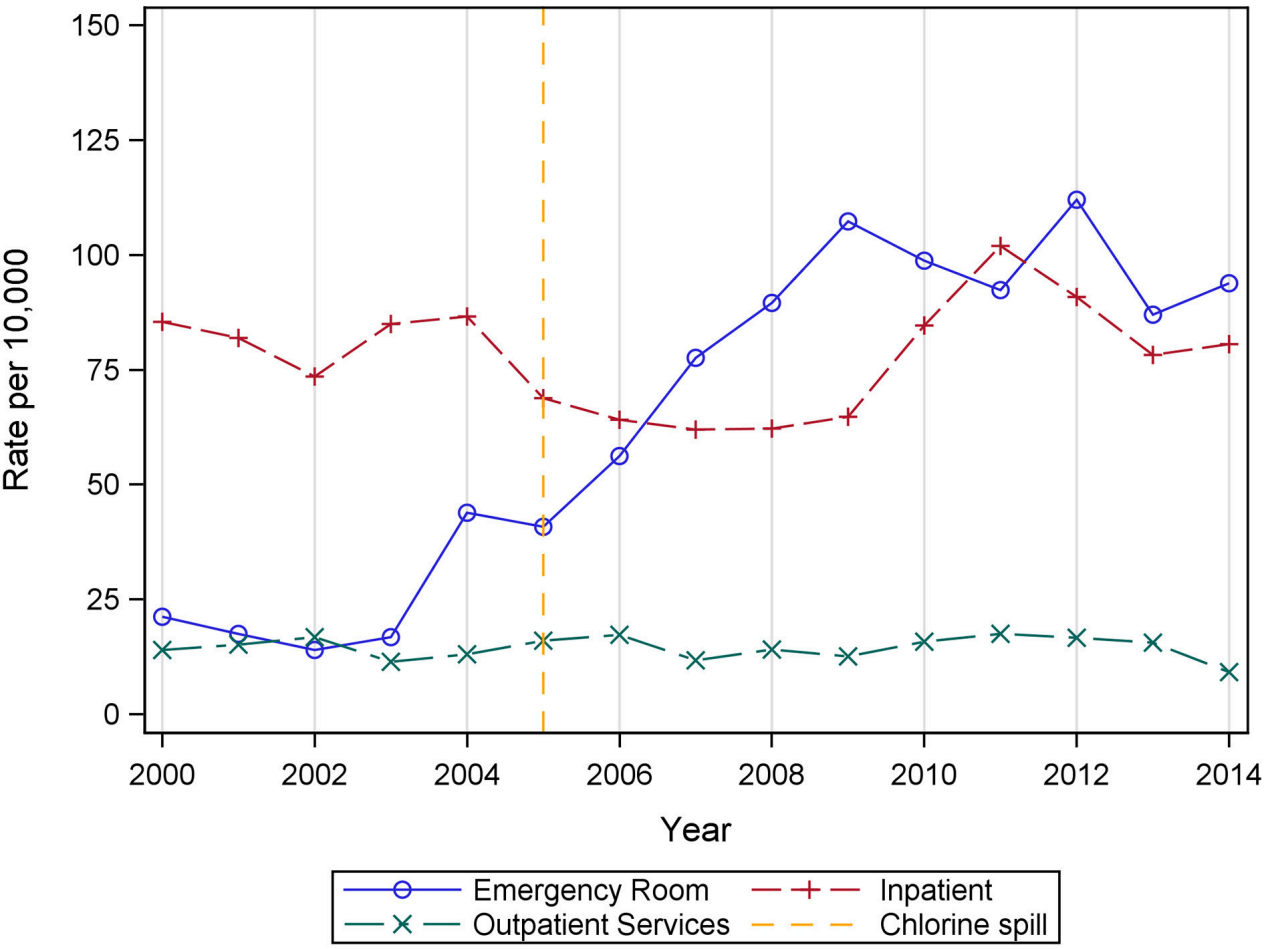
Annual rates of hospital discharges with a primary or secondary diagnosis of hypertension by visit type among Graniteville-area residents, 2000–2014.

Examination of annual discharge rates by diagnosis and visit type (Figure 2, dashed line represents the year of the chlorine disaster) showed the upward trend in hypertension-related discharges is largely attributable to emergency room visits. Emergency room discharge rates for hypertension more than doubled from 43.9 per 10,000 in 2004 to a high of 112.0 per 10,000 in 2012. A concurrent decrease was seen in CHD inpatient discharges, from 64.7 per 10,000 in 2004 to a low of 31.0 per 10,000 in 2013 (Figure S1, Supplementary Material). No notable changes in discharge rates by visit type were apparent among AMI-related hospital visits, as very few visits occurred in an outpatient or emergency room setting (Figure S2, Supplementary Material).

When comparing the time periods before the chlorine spill (2000–2004) and after the event (2005–2014), the mean annual rates of hypertension associated discharges increased from 119.3 to 176.0 per 10,000 people (Table 2). In contrast, coronary heart disease and acute myocardial infarction-related discharges decreased in the years following the chlorine gas release, from 103.4 to 76.0 per 10,000 people and 19.4 to 13.5 per 10,000 people, respectively. This pattern held when stratified by sex, but a slightly different picture emerged when pre- and post-spill admission rates were examined by age group. For people 45 years of age and older, the overall trends persisted: mean annual hypertension-related discharge rates increased from the pre-spill period to the post-spill period, while CHD and AMI discharge rates decreased. However, shifts in the rates of cardiovascular discharges were more heterogeneous for Graniteville area residents under age 45. Among those under age 25, 25–34, and 35–44 years, most discharge rates either increased or remained the same for all three diagnoses, except for AMI in 25–34 year olds which decreased slightly from 2.9 to 2.7 discharges per 10,000 persons (Table 2).

**Table 2:** Mean annual rates (per 10,000 population) of cardiovascular hospital discharges for Graniteville area residents by diagnosis, gender, and age for the time period leading up to (2000–2004) and following (2005–2014) the chlorine gas release.

	**Hypertension**		**Coronary heart disease**		**Acute myocardial infarct**	
	**Pre-spill**	**Post-spill**	**Pre-spill**	**Post-spill**	**Pre-spill**	**Post-spill**
**TOTAL, RATE (SD)**						
	119.3 (14.8)	176.0 (30.9)	103.4 (10.4)	76.0 (10.9)	19.4 (2.0)	13.5 (2.4)
**BY SEX, RATE (SD)**						
Female	131.3 (13.9)	195.5 (35.6)	83.6 (8.3)	64.3 (9.0)	17.7 (1.8)	11.5 (2.0)
Male	106.0 (16.0)	154.8 (28.1)	125.0 (14.6)	88.7 (13.6)	21.3 (3.6)	15.7 (3.4)
**BY AGE, RATE (SD)**						
≤24	3.5 (1.9)	7.3 (2.6)	–	0.6 (0.0)	–	–
25–34	28.6 (7.7)	70.8 (22.9)	5.7 (2.7)	7.1 (4.5)	2.9 (1.3)	2.6 (1.2)
35–44	68.0 (12.0)	156.6 (28.3)	29.1 (9.8)	29.1 (13.3)	7.8 (2.2)	8.4 (4.5)
45–54	158.4 (26.0)	267.4 (58.5)	117.4 (33.3)	88.3 (12.9)	19.3 (7.5)	20.7 (5.8)
55–64	267.7 (23.9)	304.7 (73.6)	245.8 (38.5)	146.3 (28.4)	40.4 (11.3)	21.1 (11.0)
65–74	343.3 (31.0)	431.7 (88.4)	405.1 (36.7)	263.1 (43.7)	64.8 (5.8)	33.3 (5.8)
75–84	470.5 (92.6)	500.0 (45.7)	459.7 (40.1)	308.5 (85.3)	87.0 (14.5)	52.2 (21.4)
≥85	493.2 (231.5)	545.8 (135.6)	331.5 (140.2)	241.7 (67.0)	142.5 (61.7)	78.0 (15.2)

*Pre-spill rates are calculated using 2000 census data, post-spill rates are calculated using 2010 census data*.

Results of the linear regressions for time series data indicated that the post-spill time period was associated with a significant increasing trend for hypertension-related hospital discharges, 8.2 for 2005–2014 vs. 5.2 for 2000–2004 (Table 3). The pre-spill time period was not associated with a statistically significant trend. Hospital discharges with CHD diagnoses were statistically significantly decreasing over time, and the slope of the regression model was steeper in the pre-spill period (−4.8, *p* = 0.007) compared to the post-spill years (−3.2, *p* < 0.001). Rates of AMI hospital discharges also decreased over the pre-spill time period (−1.5, *p* = 0.03), but the slope for the post-chlorine spill period was not statistically significant (*p* = 0.14).

**Table 3:** Results of the linear regression analyses for annual rates of cardiovascular hospitalizations by diagnosis for the years leading up to the chlorine spill (2000–2004) and the years following the spill (2005–2014).

	**Pre-spill (2000–2004)**		**Post-spill (2005–2014)**	
	**Slope**	***P*-value**	**Slope**	***P*-value**
Hypertension	5.2	0.28	8.2	<0.01
Coronary heart disease	−4.8	0.01	−3.2	<0.01
Acute myocardial infarction	−1.5	0.03	−0.3	0.14

## Discussion

We assessed three cardiovascular outcomes following a rare mass chlorine exposure event. Based on our analysis of administrative records, we were able to establish a temporal relationship between the chlorine spill and an increase in hypertension-related hospital discharges among residents of the area most impacted by the chlorine gas plume. However, due to the de-identified nature of our data source, we cannot say with any certainty whether the individuals who visited the hospital were also those exposed to chlorine. Future research is needed to clarify the etiology of the observed trend. To that end, we suggest three factors that may have contributed to the rise in hypertension hospitalizations and warrant attention: disaster-related hypertension, insufficient access to healthcare services, and chlorine-induced vascular damage.

The significant increasing trend in hypertension-related hospital discharges for the years 2005–2014 following the Graniteville train derailment and chlorine spill was surprising. Within our study population, concurrent changes in CHD and AMI hospital discharge rates were in the opposite direction. Furthermore, the upward trajectory contrasts with national trends in the prevalence of self-reported hypertension, which remained unchanged from 1999 to 2014, and in the number of inpatient discharges with a primary diagnosis of high blood pressure, which also remained static from 2000 to 2010 (26, 27). Within South Carolina, there was a minor increase in hypertension prevalence, from 28.8% in 2000 to 31.7% in 2009. But the incidence of heart disease hospitalizations has steadily declined over the same period, as have hospital visits for myocardial infarctions, specifically (28). It is important to note that the increasing trend cannot be attributed to an aging population because the demographic composition of the study population remained largely unchanged from 2000 to 2010 (Table 4), and within our dataset, the highest increases in hospital discharges following the chlorine spill were observed in younger age groups (<45 years).

**Table 4:** Age, sex, and racial composition of the raniteville study population from the 2000 and 2010 censuses.

	**Proportion of total population**	
	**2000**	**2010**
**AGE**		
Under 25	0.36	0.34
25–34	0.13	0.12
35–44	0.15	0.12
45–54	0.13	0.14
55–64	0.09	0.12
65–74	0.08	0.08
75–84	0.05	0.05
85 and Older	0.02	0.02
**SEX**		
Male	0.47	0.47
Female	0.53	0.53
RACE^*^		
White	0.65	0.64
Black	0.34	0.33
Native American or Alaskan	0.01	0.01
Asian	0.01	0.01
Hawaiian or Pacific Islander	<0.01	<0.01
Other	0.01	0.03

* *Responses could include one or two races*.

Limited access to healthcare services may have contributed to the observed rise in hypertension emergency room and hospital discharges. Within the hypertension diagnosis group, the greatest increase was seen in emergency department visits. Healthcare systems already taxed beyond capacity treating an influx of acute injuries during a disaster may be further overwhelmed by patients with exacerbated chronic conditions in the weeks and months following the event (29, 30). The phenomenon, known as a secondary surge, is consistent with descriptions of overbooked providers and insufficient medical resources in Graniteville during the recovery period (31). Further compounding the situation, the Avondale textile mill closed <2 years after the disaster, leaving more than 1,600 workers and their families without income or health insurance (32).

Disaster-related hypertension may also be independently influencing the observed trends in Graniteville cardiovascular hospital discharges. While the delayed onset of the increase is inconsistent with findings from studies conducted by Swerdel et al. and Nakamura et al., who noted an immediate increase in cardiovascular hospital discharges following Hurricane Sandy and the 2011 Japanese earthquake and tsunami, respectively (33, 34), other researchers have observed that cardiovascular effects of disasters may persist much longer. Aoki et al. found an increase of weekly occurrences of heart failure, acute coronary syndrome, stroke, and cardiopulmonary arrest in the three years following the Great East Japan Earthquake(17), Peters et al. observed a 3-fold increase in AMI incidence in the 6 year period following Hurricane Katrina (19), and Ohira et al. saw changes to blood pressure persisted for years among Japanese evacuees affected by the 2011 earthquake and tsunami (35). Although the specific biological mechanisms responsible for adverse cardiovascular symptoms following disasters are not fully understood, the effects may be further compounded by heightened susceptibility to vascular pathologies introduced by chlorine exposure among Graniteville residents.

Another factor which may be contributing to the unusual increase in hypertension-related hospital discharges is chlorine-induced systemic vascular damage among Graniteville residents exposed to the spill. As a member of the highly reactive halogen elements, chlorine readily forms hydrochloric (HCl) and hypochloric (HOCl) acids and oxygen free radicals when it comes into contact with water in the mucous membranes and alveolar spaces (11). The hydrolytic products exhaust stores of antioxidants, activate pro-inflammatory cells, and interfere with cell signaling (10, 36). As an example of the latter, chlorine has been shown to interfere with endothelial nitric oxide synthase (eNOS)-mediated signaling in animal models (37). Nitric oxide produced by eNOS regulates vasodilation and is responsible for maintaining vascular homeostasis; so disruption of this pathway results in increased susceptibility to hypertension and inflammation (37, 38). A second potential mechanism for chlorine-induced vascular disease is attributable to one of the products of chlorine's hydrolysis, HOCl. Hypochloric acid is known to react with low- and high-density lipoproteins (LDL and HDL) converting the molecules into pro-atherogenic compounds (39). Heightened vulnerability to chronic vascular dysfunction instigated by chlorine exposure may explain why the rise of related hospital discharges peaked years after the event. While the damage to lung epithelial tissue occurs within minutes of exposure, the systemic diffusion of pro-inflammatory cells and secondary reactive oxygen and chlorine species prolongs the physiological damage and may initiate biological processes with long-term health consequences (38).

Our study has important clinical significance. Elevated blood pressure may be involved with the risks associated with lung function and cardiovascular disease. Numerous population studies have shown that airflow restriction (as measured by forced expiratory volume in 1 s (FEV 1) or the FEV 1/forced vital capacity ratio) is a predictor of future risk of hypertension (40). Further, chronic obstructive pulmonary disease (COPD) has been associated with cardiovascular events (41), congestive heart failure, ischemic heart disease, arrhythmia, stroke, hypertension, and peripheral artery disease (42). This association is supported by research which demonstrates that airflow limitation is a predictor of future risks of hypertension and cardiovascular events (43). The 2017 American College of Cardiology (ACC)/American Heart Association (AHA) Guideline for the Prevention, Detection, Evaluation, and Management of High Blood Pressure in Adults, which provides an evidence-based approach to reduce CVD risk through lowering of blood pressure, redefined the lower threshold for systolic blood pressure to a target of <130 mm Hg to reduce the risk of several important outcomes including risk of myocardial infarction, stroke, heart failure, and major cardiovascular events(44, 45). The implications for a high risk population such as Graniteville is that these recommendations increase the number of individuals at risk from the lung effects on elevated blood pressure.

Our study also points toward an important public health problem. Individuals in disadvantaged communities are unlikely to receive regular routine health care and long-term follow up. This allows slowly developing but potentially life-threatening problems, such as hypertension, to go undetected for long periods of time. Even if such a problem is discovered, the individual may not be followed on a regular basis to ensure adequate long-term control. The lack of routine care also increases the probability that individuals in such communities will not be referred for mental health care if they develop symptoms of anxiety, depression or post-traumatic stress after a disaster. At the least, an effort to regularly assess blood pressures among potentially affected residents and to provide appropriate treatment referrals would likely be highly beneficial at relatively modest cost.

To our knowledge, this is the first study to examine temporal trends in cardiovascular hospitalizations following a mass chlorine exposure event. Use of a comprehensive administrative data set that included hospital discharges from other states in addition to South Carolina is a strength of our study. We were also able to avoid the need for extensive information on covariates with the use of a quasi-experimental interrupted time series design.

There are also limitations to this study As discussed earlier, we cannot say with certainty whether the individuals who visited the hospital were also those who were exposed to the chlorine gas plume or were directly impacted by the resulting disaster effects on the community. Further research is needed to determine if this is the case, and if so, what are the biologic mechanisms underlying the association. Additionally, our population-level design cannot assess disease etiology at an individual level, only at the population level, and we cannot discern which of disaster risk factors best explained the observed effects on CVD or whether their effects were synergistic. Further study is needed to assess whether these observed effects were caused by chlorine exposure, disaster-related stress, impaired healthcare services, and/or some yet unmeasured risk factor. Other limitations of our study design include limited statistical power and the potential for misclassification. Because our data was aggregated by year, there were only five time points available for the “pre-spill” period. Addresses used to select our study population were provided by patients and may not have been the address where the patient was located during the chlorine disaster. Additionally, only the year of discharge was available to us, so the “post-spill” time period included 5 days of “unexposed” time.

## Conclusion

The utility of chlorine in diverse household and industrial applications ensures the chemical's ubiquity will persist. With domestic production of chlorine nearing 10 million metric tons annually, there continues to be real concern for another large industrial or transport accident (46). Indeed, the Department of Homeland Security included chlorine in one of its 15 “National Planning Scenarios” and lists chlorine among the “chemicals of concern” in the Chemical Facility Anti-Terrorism Standards (6 CFR Part 27) (47–49). Therefore, it would be prudent to improve our understanding of the persistent health effects from chlorine exposure so that we can be better prepared for future incidents involving chlorine.

Our study suggests that chlorine itself or stress associated with exposure to a chlorine disaster may be associated with later development of hypertension. Further research is needed to determine whether the residents of Graniteville exposed to chlorine or the resulting traumatic stress are at an increased risk for hypertension or other cardiovascular events, and if so, to elucidate the physiological processes responsible for systemic vascular damage. Nevertheless, this study is a small step toward identifying and characterizing an association that has the potential to cause considerable excess morbidity and costs to our healthcare system should another large-scale release of chlorine occur in the future.

## Ethics Statement

This study was reviewed and approved by the Medical University of South Carolina Institutional Review Board and the South Carolina Revenue and Fiscal Affairs data oversight committee. Before receipt, all data were de-identified by the SC RFA, therefore the requirement of informed consent from individual subjects was waived by the IRB. To further protect the privacy of area residents, the data presented are in aggregate form.

## Author Contributions

AH, JV, LI, and ES conceived and designed the study with statistical consultation with BC and AL. AH performed the analysis and took the lead in writing the manuscript with support from ES and JV. DL and ES also contributed to the interpretation of the results.

### Conflict of Interest Statement

The authors declare that the research was conducted in the absence of any commercial or financial relationships that could be construed as a potential conflict of interest.

## Supplementary Material

Figure S1 Annual rates of hospital discharges with a primary or secondary diagnosis of coronary heart disease by visit type among Graniteville-area residents, 2000–2014.

Figure S2 Annual rates of hospital discharges with a primary or secondary diagnosis of acute myocardial infarction by visit type among Graniteville-area residents, 2000–2014.
