# Supplementary figures and images for: Temporal Trends in Cardiovascular Hospital Discharges Following a Mass Chlorine Exposure Event in Graniteville, South Carolina
Source: Front Public Health. 2019 May 8;7:112. doi: 10.3389/fpubh.2019.00112 (PMC6517492; doi:10.3389/fpubh.2019.00112)

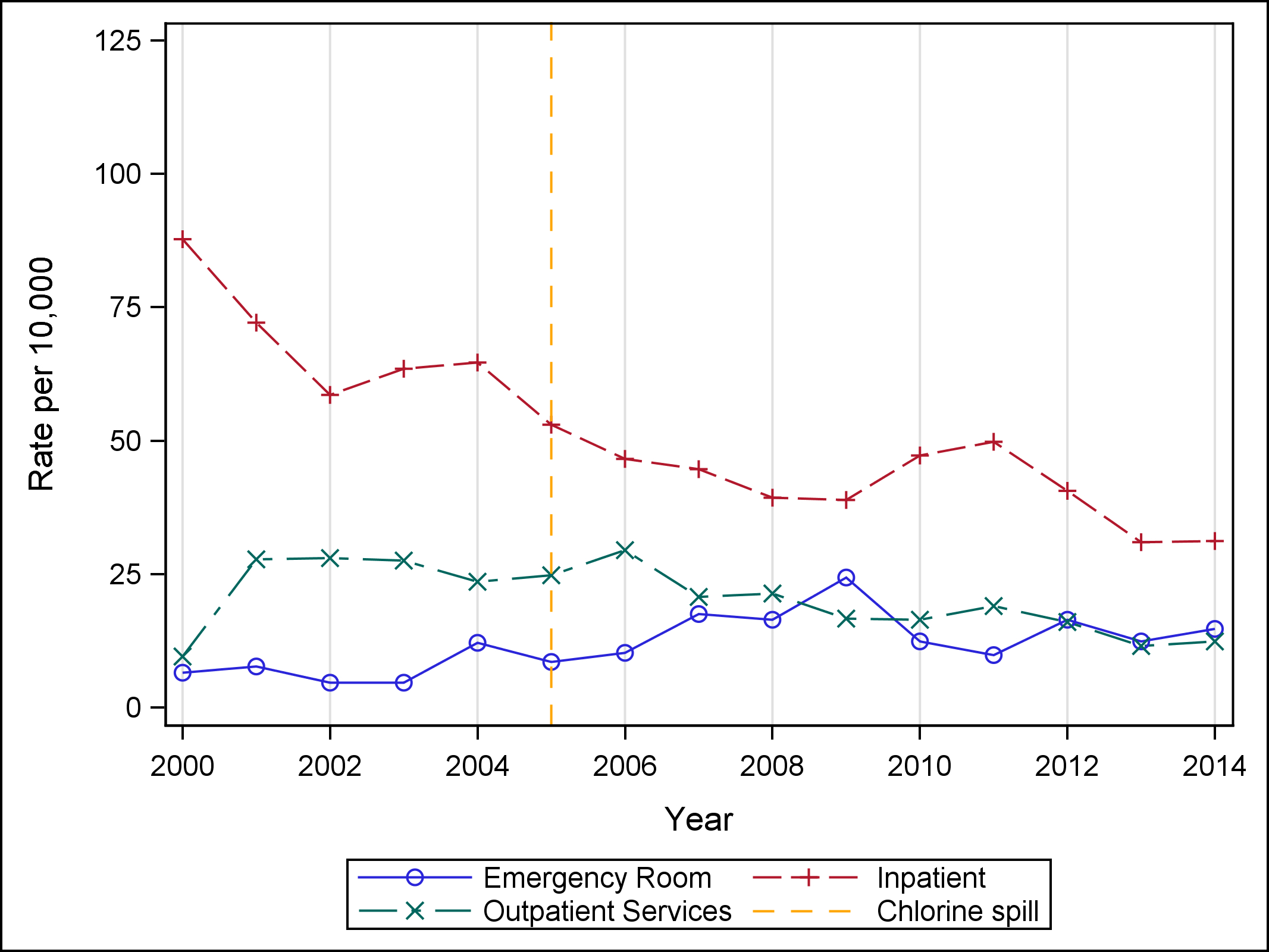

Supplement: Figure S1 — Annual rates of hospital discharges with a primary or secondary diagnosis of coronary heart disease by visit type among Graniteville-area residents, 2000–2014. [file Image_1.jpg]

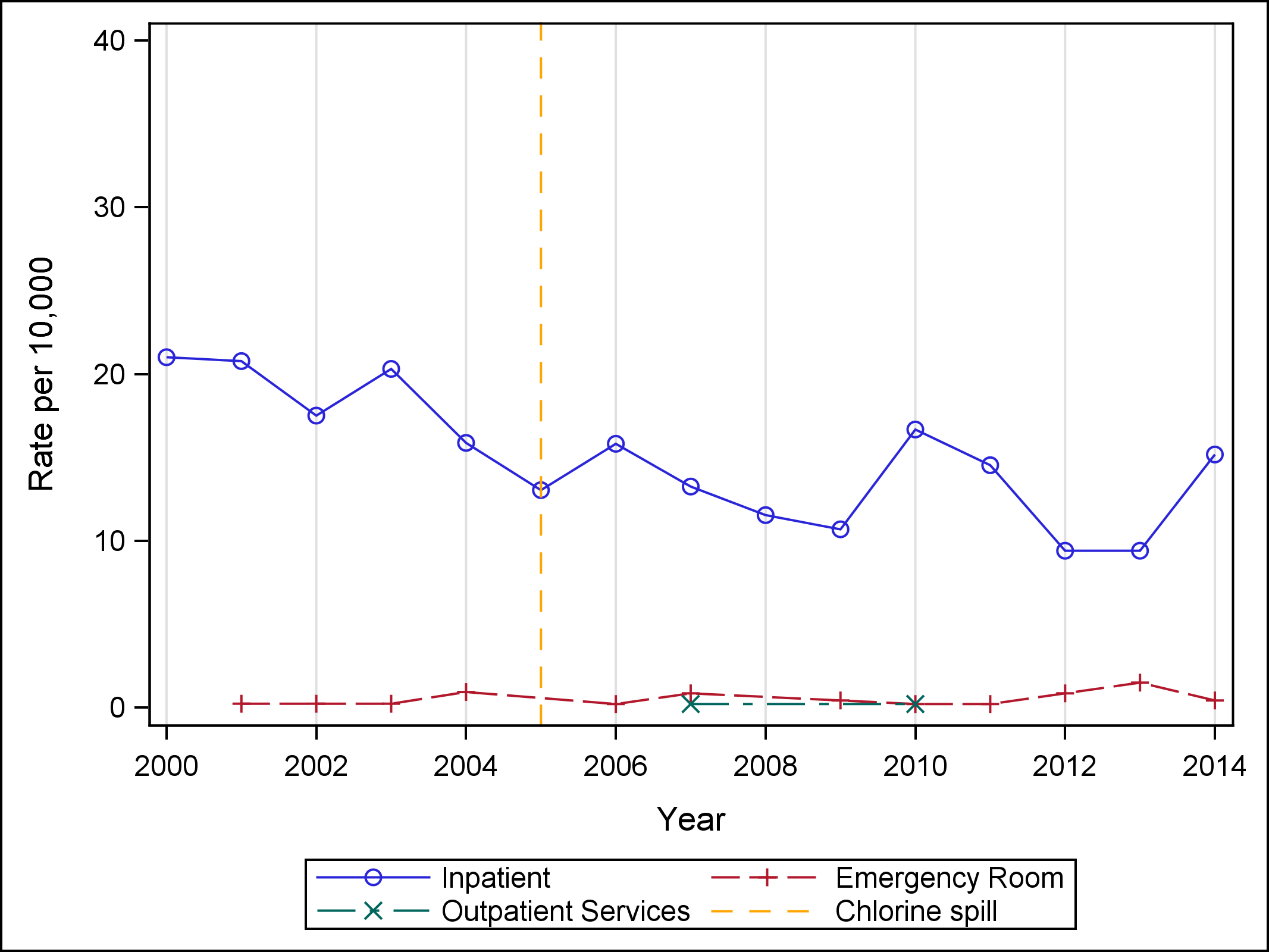

Supplement: Figure S2 — Annual rates of hospital discharges with a primary or secondary diagnosis of acute myocardial infarction by visit type among Graniteville-area residents, 2000–2014. [file Image_2.jpg]
